# TGF-beta1 regulates human brain pericyte inflammatory processes involved in neurovasculature function

**DOI:** 10.1186/s12974-016-0503-0

**Published:** 2016-02-11

**Authors:** Justin Rustenhoven, Miranda Aalderink, Emma L. Scotter, Robyn L. Oldfield, Peter S. Bergin, Edward W. Mee, E. Scott Graham, Richard L. M. Faull, Maurice A. Curtis, Thomas I-H. Park, Mike Dragunow

**Affiliations:** Department of Pharmacology and Clinical Pharmacology, The University of Auckland, Auckland, 1023 New Zealand; Department of Anatomy, The University of Auckland, Auckland, 1023 New Zealand; Centre for Brain Research, The University of Auckland, Auckland, 1023 New Zealand; Lab Plus, Auckland, 1023 New Zealand; Auckland City Hospital, Auckland, 1023 New Zealand

**Keywords:** Phagocytosis, Inflammation, Cytokine, Chemokine, BBB, Alzheimers, NOX4, MMP-2, IL-6, SMAD2/3

## Abstract

**Background:**

Transforming growth factor beta 1 (TGFβ_1_) is strongly induced following brain injury and polarises microglia to an anti-inflammatory phenotype. Augmentation of TGFβ_1_ responses may therefore be beneficial in preventing inflammation in neurological disorders including stroke and neurodegenerative diseases. However, several other cell types display immunogenic potential and identifying the effect of TGFβ_1_ on these cells is required to more fully understand its effects on brain inflammation. Pericytes are multifunctional cells which ensheath the brain vasculature and have garnered recent attention with respect to their immunomodulatory potential. Here, we sought to investigate the inflammatory phenotype adopted by TGFβ_1_-stimulated human brain pericytes.

**Methods:**

Microarray analysis was performed to examine transcriptome-wide changes in TGFβ_1_-stimulated pericytes, and results were validated by qRT-PCR and cytometric bead arrays. Flow cytometry, immunocytochemistry and LDH/Alamar Blue® viability assays were utilised to examine phagocytic capacity of human brain pericytes, transcription factor modulation and pericyte health.

**Results:**

TGFβ_1_ treatment of primary human brain pericytes induced the expression of several inflammatory-related genes (*NOX4*, *COX2*, *IL6* and *MMP2*) and attenuated others (*IL8*, *CX3CL1*, *MCP1* and *VCAM1*). A synergistic induction of IL-6 was seen with IL-1β/TGFβ_1_ treatment whilst TGFβ_1_ attenuated the IL-1β-induced expression of CX3CL1, MCP-1 and sVCAM-1. TGFβ_1_ was found to signal through SMAD2/3 transcription factors but did not modify nuclear factor kappa-light-chain-enhancer of activated B cells (NF-kB) translocation. Furthermore, TGFβ_1_ attenuated the phagocytic ability of pericytes, possibly through downregulation of the scavenger receptors CD36, CD47 and CD68. Whilst TGFβ did decrease pericyte number, this was due to a reduction in proliferation, not apoptotic death or compromised cell viability.

**Conclusions:**

TGFβ_1_ attenuated pericyte expression of key chemokines and adhesion molecules involved in CNS leukocyte trafficking and the modulation of microglial function, as well as reduced the phagocytic ability of pericytes. However, TGFβ_1_ also enhanced the expression of classical pro-inflammatory cytokines and enzymes which can disrupt BBB functioning, suggesting that pericytes adopt a phenotype which is neither solely pro- nor anti-inflammatory. Whilst the effects of pericyte modulation by TGFβ_1_ in vivo are difficult to infer, the reduction in pericyte proliferation together with the elevated IL-6, MMP-2 and NOX4 and reduced phagocytosis suggests a detrimental action of TGFβ_1_ on neurovasculature.

**Electronic supplementary material:**

The online version of this article (doi:10.1186/s12974-016-0503-0) contains supplementary material, which is available to authorized users.

## Background

Neuroinflammation contributes to the development and progression of epilepsy [1], traumatic brain injuries [2], stroke [3] and many neurodegenerative diseases [4]. Inflammatory cytokines are key mediators of the brain’s immune response and associated neurodegeneration [5]. Transforming growth factor beta (TGFβ) is a pleiotropic cytokine in the brain with roles in regulating cell proliferation, differentiation, survival and scar formation [6–9]. Furthermore, TGFβ orchestrates both pro- and anti-inflammatory responses in a cell and context-dependent manner [10–12]. TGFβ exists in three isoforms (TGFβ_1–3_), of which TGFB_1_ was the first identified and is the most widely studied. TGFβ exerts its actions through the serine/threonine kinase receptors, transforming growth factor beta receptor 1 (TGFBR1), TGFBR2 and TGFBR3. Activation of TGFBRs by TGFβ ligand binding initiates a signal transduction pathway predominantly through SMAD transcription factors [13].

Several cell types in the central nervous system (CNS) are capable of producing TGFβ_1_. Endothelial cells secrete this growth factor, and this is enhanced by co-culture with pericytes [14, 15]. Following brain injury, microglia and astrocytes produce large concentrations of TGFβ_1_ [8, 16, 17], whilst glioblastoma multiforme enhances TGFβ_1_ expression, possibly due to the increased and abnormal angiogenesis [18–20]. Furthermore, TGFβ_1_ is elevated in several inflammatory conditions including Alzheimer’s disease, type 1 diabetes mellitus and acute brain injuries such as ischaemic stroke [21–24].

A number of immunologically active brain cells express TGFBRs and can therefore respond to secretions of this growth factor. Microglia are the brain’s predominant immune cell and the most widely studied with respect to their inflammatory response [25]. TGFβ_1_ promotes a strong anti-inflammatory phenotype in microglia through attenuated cytokine, chemokine, adhesion molecule and reactive oxidative species (ROS) production; however, it has no effect on human leukocyte antigen (HLA) antigen presentation complexes [10, 26–28]. Furthermore, TGFβ_1_ enhances microglial phagocytosis of amyloid beta, thereby reducing plaque burden [29]. Astrocytes show a more complex regulation of immune responses by TGFβ_1_ stimulation, with reports of both enhancement and inhibition of chemokine and cytokine production [11, 12, 30, 31]. Other cells, including those derived from the choroid plexus and leptomeninges, also display attenuated inflammatory responses with TGFβ_1_ treatment [27, 32].

Previous work in our lab using human brain pericytes demonstrated a reduction in interferon gamma (IFNγ)-induced HLA-DR, HLA-DP and HLA-DQ expression with TGFβ_1_ treatment, suggesting an anti-inflammatory role [27]. Pericytes are multifunctional cells that surround endothelial cells and contact numerous parenchymal brain cells including astrocytes, neurons and microglia to form a neurovascular unit [33]. Pericyte coverage of brain vasculature is vital with respect to blood-brain barrier (BBB) formation and maintenance [34–36]. Several labs, including our own, have shown that pericytes can contribute to the brain’s immune response through cytokine, chemokine and adhesion molecule expression and that they display phagocytic potential [37–42].

The function of TGFβ_1_ on pericytes has been briefly studied, however, largely with respect to differentiation, proliferation or angiogenesis [43, 44]. Little is known about the role of TGFβ_1_ in the context of human brain pericyte immune function. We therefore sought to investigate how TGFβ_1_ modifies the human brain pericyte inflammatory response.

## Methods

### Tissue source

Human middle temporal gyrus brain tissue was obtained, with informed consent, from surgeries of patients with drug-resistant temporal lobe epilepsy. All specimens were collected with written patient consent. All protocols used in this study were approved by the Northern Regional Ethics Committee (New Zealand), and all methods were carried out in accordance with the approved guidelines.

### Mixed glial cultures isolated from human brain tissue

Mixed glial cultures containing astrocytes, pericytes and microglia were isolated from adult human brain tissue with minor modifications as described previously [45]. As per [42], cells were grown until passage five before use in order to dilute out non-proliferating microglia and astrocytes and obtain a pure pericyte population. Pericyte cultures at passages five-eight were used for all experiments. Detailed characterisation of pericyte cultures has been performed previously [37, 42].

### Cytokine and drug treatments

To examine the effect of cytokines on pericyte responses, cells were treated with 0.1–10 ng/mL TGFβ_1_ (PeproTech, NJ, USA; vehicle, 1 mM citric acid, pH 3 with 0.1 % bovine serum albumin (BSA)) or interleukin 1 beta (IL-1β; Peprotech, NJ, USA; vehicle, 0.1 % BSA in phosphate buffered saline (PBS)) for 0–72 h. Details for each experiment are noted in appropriate figure legends. To induce apoptosis as a positive control for cleaved caspase 3 (CC3) staining, pericytes were treated with 50 nM okadaic acid (Sigma-Aldrich, MO, USA) for 24 h. Cells were treated with drugs/cytokines by addition of a 1:100 dilution of a 100 × stock.

### Collection of conditioned media and cytokine measurement by cytometric bead array

Conditioned media was collected from cells grown in a 96-well plate. Media was spun at 160×*g* for 5 min to collect any detached cells or debris. Supernatant was obtained and stored at −20 °C. The concentration of cytokines was measured using a cytometric bead array (CBA; BD Biosciences, CA, USA) as per manufacturer’s instructions. CBA samples were run on an Accuri C6 flow cytometer (BD Biosciences, CA, USA). Data was analysed using FCAP-array software (version 3.1; BD Biosciences, CA, USA) to convert fluorescent intensity values to concentrations using a ten-point standard curve (0–5000 pg/mL) as described previously [46].

### Immunocytochemistry

Cells were fixed in 4 % paraformaldehyde (PFA) for 15 min and washed in PBS with 0.1 % triton X-100 (PBS-T). Cells were incubated with primary antibodies (Additional file 1: Table S1) overnight at 4 °C in immunobuffer containing 1 % goat serum, 0.2 % Triton X-100 and 0.04 % thimerosal in PBS. Cells were washed in PBS-T and incubated with appropriate anti-species fluorescently conjugated secondary antibodies overnight at 4 °C. Cells were washed again and incubated with Hoechst 33258 (Sigma-Aldrich, MO, USA) for 20 min. Images were acquired at ×10 magnification using the automated fluorescence microscope ImageXpress® Micro XLS (version 5.3.0.1, Molecular Devices, CA, USA). Quantitative analysis of intensity measures and positively stained cells was performed using the Cell Scoring and Show Region Statistics analysis modules on MetaXpress® software (version 5.3.0.1, Molecular Devices, CA, USA).

### Phagocytosis assays

To evaluate phagocytosis by microscopy, cells were treated with 0–10 ng/mL TGFβ_1_ for 24 h, followed by a further 24-h incubation with Fluoresbrite® YG carboxylate microspheres of 1 μm diameter (Polysciences Inc, PA, USA; 1:1000 dilution) at 37 °C, 5 % CO_2_. At completion, cells were washed twice with PBS to remove un-phagocytosed beads and fixed in 4 % PFA as per immunocytochemistry. Nuclear staining was visualised by a 30-min incubation with the DNA-specific dye DRAQ5 (BioStatus, UK). Images were obtained using the ImageXpress® Micro XLS microscope and the percentage of phagocytic cells determined using the Cell Scoring module on MetaXpress® software.

To evaluate phagocytosis by flow cytometry, cells were treated with 0–10 ng/mL TGFβ_1_ for 24 h, followed by a further 2-h incubation with Fluoresbrite® YG carboxylate microspheres of 1-μm diameter (1:1000 dilution) at 37 °C, 5 % CO_2_. At completion, cells were washed twice with PBS, and 0.25 % trypsin-ethylenediaminetetraacetic acid (EDTA) was added to remove beads bound to the cell surface and bring cells into suspension. Selected samples were incubated for 10 min with 7-aminoactinomycin D (7-AAD; BD Biosciences, CA, USA) to assess viability. Samples were run on an Accuri C6 flow cytometer and viable cells gated based on forward scatter and side scatter. Mean fluorescent intensity (MFI) of the live cells was detected, indicative of the quantity of beads internalised.

### Confocal laser scanning microscopy

Cells destined for confocal microscopy were plated at 5000 cells/well on 8-mm #1.5 glass coverslips (Menzel Gläser, Germany) within a 48-well plate. Fluoresbrite® YG carboxylate microspheres of 1-μm diameter (1:10,000 dilution) were added to cells for 24 h at 37 °C, 5 % CO_2_ and at completion washed twice in PBS to remove un-phagocytosed beads. Cells were fixed in 4 % PFA and immunostained for platelet-derived growth factor receptor beta (PDGFRβ) as per immunocytochemistry, with the exception of diluting primary and secondary antibodies in donkey immunobuffer (1 % donkey serum, 0.2 % Triton X-100 and 0.04 % thimerosal in PBS). Coverslips were mounted onto glass slides using fluorescent mounting medium (DAKO, Denmark). Confocal images were acquired using an oil immersion lens (×63 magnification, 1.4NA) in a Z-series with a gap of 0.8 μm using a Zeiss LSM 710 inverted confocal microscope (Biomedical Imaging Research Unit, University of Auckland) with ZEN 2010 software (Carl Zeiss, Germany).

### EdU proliferation assay

5-Ethynyl-2′-deoxyuridine (EdU; 10 μM) was added to pericyte cultures 24 h prior to completion of experiment. Cells were fixed in 4 % PFA for 15 min and EdU visualised as per manufacturer’s instructions (Life Technologies, CA, USA). Cell nuclei were counterstained with Hoechst 33258 as per immunocytochemistry.

### Alamar Blue® viability assay

To determine cell viability, Alamar Blue® (1:10 final dilution in culture medium; Life Technologies, CA, USA) was added to cells in a 96-well plate for 2 h at 37 °C, 5 % CO_2_. Fluorescence (Ex 544/ Em 590) was read on a FLUOStar Optima plate reader (BMG Labtech, Germany). Background fluorescence of wells with media but no cells was subtracted from all values, and fluorescence intensity values were normalised to cell number using imaging of Hoechst positive cells. Fluorescence intensity values were then normalised to vehicle control.

### LDH cytotoxicity assay

To determine cellular toxicity, extracellular lactate dehydrogenase (LDH) was detected as per manufacturer’s instructions (Roche, Switzerland). Briefly, a positive control was first prepared by lysing cells through addition of DMEM/F12 media containing 1 % Triton X-100. The culture media from all samples was then transferred to a new plate and centrifuged at 160×*g* for 10 min at room temperature. To a new 96-well plate, 50 μL of centrifuged media was transferred and 50 μL of LDH reaction mixture was added. The plate was incubated at room temperature for 30 min and absorbance measured at 540 nm on a FLUOStar Optima plate reader. Absorbance of culture media containing no cells was subtracted from all values and the absorbance normalised to that of the positive control (100 % cytotoxicity).

### Quantitative real-time reverse transcriptase PCR

Cells destined for quantitative real-time reverse transcriptase PCR (qRT-PCR) were plated at 100,000 cells/well in a six-well plate. At completion of the experiment, cells were washed in PBS and RNA extraction, and purification was performed using the RNeasy® mini kit (Qiagen, Netherlands) as per manufacturer’s instructions from duplicate wells. RNA was treated with DNase I (1 μg DNase I/1 μg RNA) using the RQ1 RNase-free DNAse kit (Promega, WI, USA) and cDNA made using the Superscript® III First-Strand Synthesis kit (Life Technologies, CA, USA). Quantitative real-time PCR was performed using Platinum® SYBR® Green qPCR SuperMix-UDG with Rox (Life Technologies, CA, USA) on a 7900HT Fast Real-Time PCR system (Applied Biosystems, Life Technologies, CA, USA). Standard curves were run for all primers, and efficiencies were all 100 ± 10 % (Additional file 2: Table S2). Relative gene expression analysis was performed using the 2^−ΔΔCt^ method to the housekeeping gene *GAPDH*.

### Microarray

Microarray analysis of TGFβ_1_-treated pericytes was conducted on technical triplicates of a single patient donor. Cells destined for microarray were treated for 72 h with vehicle (1 mM citric acid, pH 3 with 0.1 % BSA) or 10 ng/mL TGFβ_1_. Cells were washed in PBS and lysed in 1 mL of TRIzol reagent (Life Technologies, CA, USA). To this, 200 μL of chloroform was added, and samples were vigorously shaken and centrifuged at 12,000×*g* for 15 min at 4 °C. The upper aqueous phase was transferred to a new tube and an equal volume of 70 % ethanol added. RNA purification was performed using the RNeasy® mini kit as per qRT-PCR experiments. RNA quality was analysed using a 2100 Bioanalyzer (Agilent Technologies, CA, USA) with all samples having a RIN value of 10. RNA was labelled and hybridised to Affymetrix Genechip® Primeview™ Human Gene Expression Arrays (Santa Clara, CA, USA) according to manufacturer’s instructions. Microarray was performed and analysed by New Zealand Genomics Limited (NZGL). Candidate microarray hits for qRT-PCR and CBA validation were selected based on the magnitude of change and prior evidence for involvement in neuro-immune responses but do not include all modified inflammatory genes.

### Statistical analysis

Unless otherwise stated, all experiments were performed at least three independent times on tissue from three different donors. Statistical analysis was carried out using an unpaired Student’s *t* test, one-way analysis of variance (ANOVA) with Dunnett’s multiple comparison test to compare treatments versus vehicle control, ANOVA with Newman-Keuls multiple comparison test to compare all conditions, or a two-way ANOVA to compare the effect of both time and cytokine stimulation (Graphpad Prism 5.02). For statistical analysis of qRT-PCR data, ΔCt values were used. Statistical analysis of microarray data was performed as described previously [37].

## Results

### TGFβ_1_ modifies inflammatory gene expression in human brain pericytes

**Fig. 1:**
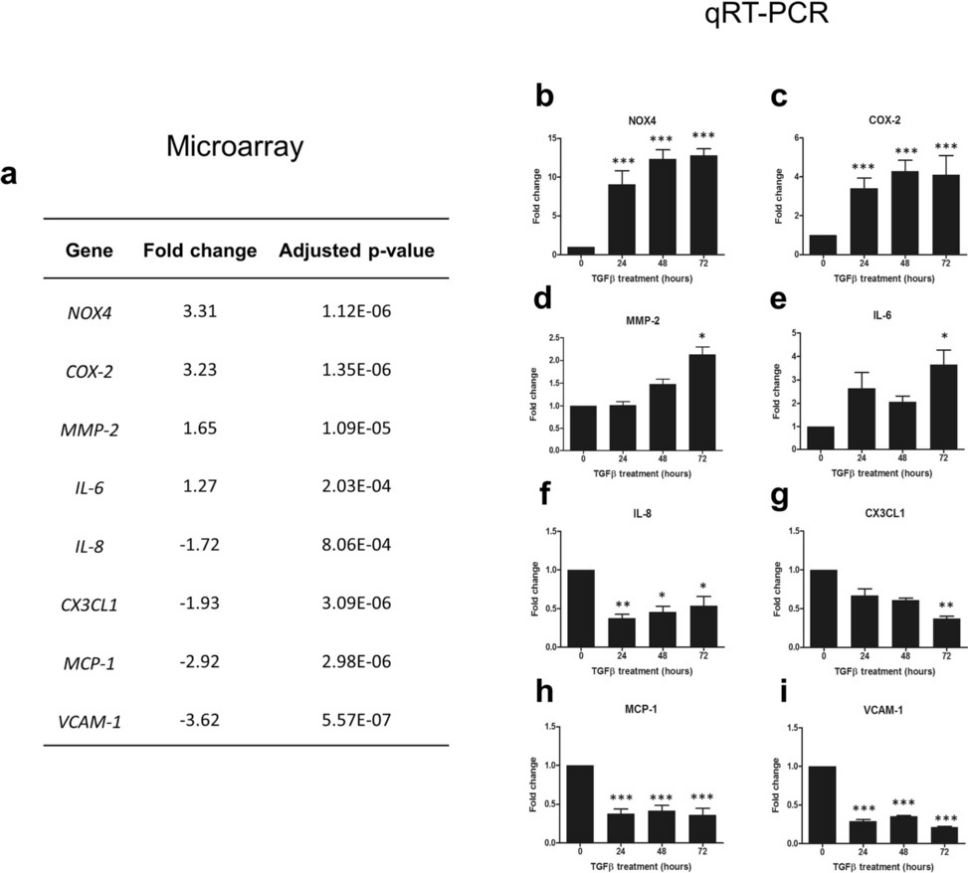
TGFβ_1_ modifies inflammatory gene expression in human brain pericytes. Primary human brain pericytes were treated with vehicle (1 mM citric acid, pH 3 with 0.1 % BSA) or 10 ng/mL TGFβ_1_ for 72 h. RNA was extracted and expression of inflammation-related genes determined by microarray (**a**). Changes in gene expression (*NOX4* (**b**), *COX2* (**c**), *MMP2* (**d**), *IL6* (**e**), *IL8* (**f**), *CX3CL1* (**g**), *MCP1* (**h**) and *VCAM1* (**i**)) were confirmed by qRT-PCR from three independent experiments with 0–72 h treatments with 10 ng/mL TGFβ_1_. *p < 0.05, **p < 0.01, ***p < 0.001 versus 0 h time point (ANOVA)

The role of TGFβ_1_ in modifying pericyte phenotype and associated functional responses including scarring, proliferation and differentiation has been previously studied [43, 44]. However, its effect on pericyte-mediated immune responses has not been well characterised. Previous work from our lab has identified a potential anti-inflammatory role for TGFβ_1_ in pericytes through prevention of IFNγ-induced HLA-DR expression [27]. In order to further understand the inflammatory phenotype adopted by TGFβ_1_-stimulated brain pericytes, microarray analysis was undertaken. As expected, several inflammatory genes showed attenuated expression, including interleukin 8 (*IL8*), *p* = 8.06^−4^; *CX3CL1*, *p* = 3.09^−6^; monocyte chemoattractant protein 1 (*MCP1*), *p* = 2.98^−6^; and vascular cell adhesion molecule 1 (*VCAM1*), *p* = 5.57^−7^ (Fig. 1a). Surprisingly, however, a number of inflammatory-related genes were also found to be induced by TGFβ_1_ treatment, including nicotinamide adenine dinucleotide phosphate-oxidase 4 (*NOX4*), *p* = 1.12^−6^; cyclooxygenase 2 (*COX2*), *p* = 1.35^−6^; matrix metalloproteinase 2 (*MMP2*), *p* = 1.09^−4^; and interleukin 6 (*IL6*), *p* = 2.03^−4^ (Fig. 1a). These changes were confirmed by qRT-PCR with TGFβ_1_-induced up-regulation of *NOX4* (*p* < 0.001; Fig. 1b), *COX2* (*p* < 0.001; Fig. 1c), *MMP2* (*p* < 0.05; Fig. 1d) and *IL6* (*p* < 0.05; Fig. 1e) and attenuation of *IL8* (*p* < 0.05; Fig. 1f), *CX3CL1* (*p* < 0.01; Fig. 1g), *MCP1* (*p* < 0.001; Fig. 1h) and *VCAM1* (*p* < 0.001; Fig. 1i) at 72 h. Furthermore, several changes were apparent as early as 24 h after TGFβ_1_ treatment (*NOX4*, *p* < 0.001, Fig. 1b; *COX2*, *p* < 0.001, Fig. 1c; *IL8*, *p* < 0.01, Fig. 1f; *MCP1*, *p* < 0.001, Fig. 1h and *VCAM1*, *p* < 0.001, Fig. 1i).

### TGFβ_1_ modifies inflammatory protein secretion in human brain pericytes

**Fig. 2:**
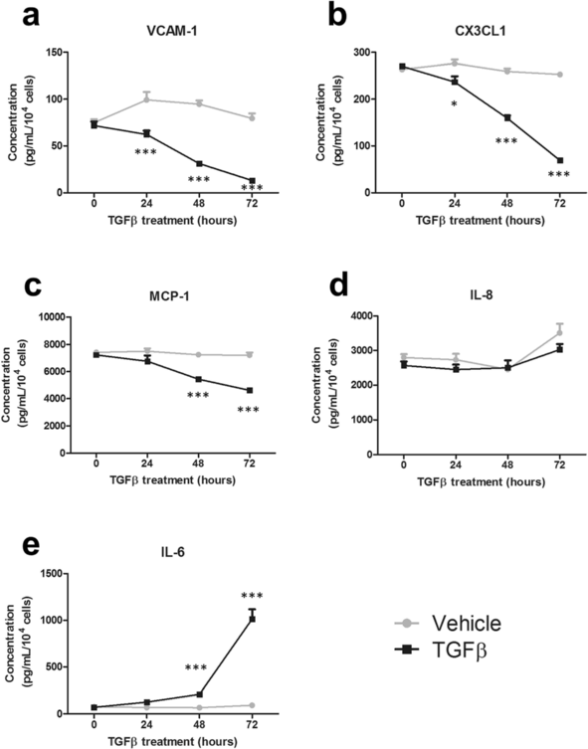
TGFβ_1_ modifies inflammatory protein secretion in human brain pericytes. Primary human brain pericytes were treated with vehicle (1 mM citric acid, pH 3 with 0.1 % BSA) or 10 ng/mL TGFβ_1_ for 0–72 h, and conditioned media was collected. Concentration of secreted proteins (sVCAM-1 (**a**), CX3CL1 (**b**), MCP-1 (**c**), IL-8 (**d**) and IL-6 (**e**)) was determined by a multiplex cytometric bead array. One representative experiment (*N* = 4 wells) from three independent experiments is shown. *p < 0.05, **p < 0.01, ***p < 0.001 versus vehicle control at each time point (two-way ANOVA)

Having identified a role for TGFβ_1_ in modifying pericyte inflammatory gene expression, we sought to ascertain whether this translated to a functional change in protein levels. Several of the genes affected by TGFβ_1_ are secreted cytokines, chemokines or cleavable/secreted adhesion molecules. We therefore determined the concentration of these proteins in conditioned media from TGFβ_1_-treated pericytes. All tested inflammatory mediators were found to be secreted basally by pericytes and, consistent with microarray and qRT-PCR results, a 72-h treatment with TGFβ_1_ attenuated this secretion of soluble VCAM-1 (sVCAM-1; *p* < 0.001; Fig. 2a), CX3CL1 (*p* < 0.001; Fig. 2b) and MCP-1 (*p* < 0.001; Fig. 2c). Interestingly, the expression of IL-8 was found to be unchanged (*p* > 0.05, Fig. 2d). Furthermore, IL-6 secretion was significantly enhanced by TGFβ_1_ treatment (*p* < 0.001; Fig. 2e). Secretions of sVCAM-1 (*p* < 0.001; Fig. 2a) and CX3CL1 (*p* < 0.05; Fig. 2b) were significantly decreased after a 24-h treatment with TGFβ_1_, whilst MCP-1 attenuation (*p* < 0.001; Fig. 2c) and IL-6 induction (*p* < 0.001; Fig. 2e) was apparent at 48 h.

### Synergistic and preventative effects of TGFβ_1_ on IL-1β-induced inflammatory protein secretion

**Fig. 3:**
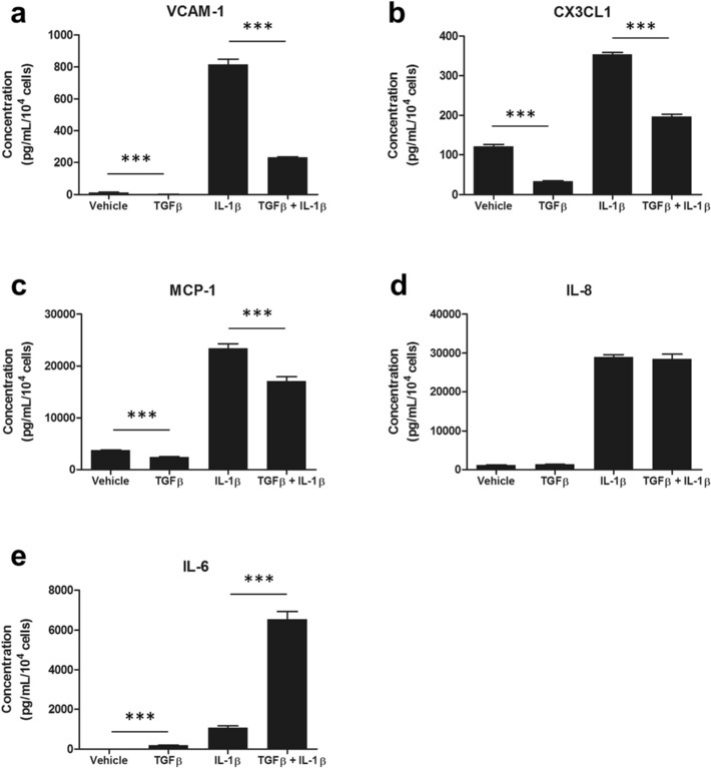
Synergistic and preventative effects of TGFβ_1_ on IL-1β-induced inflammatory protein secretion. Primary human brain pericytes were treated with vehicle (1 mM citric acid, pH 3 with 0.1 % BSA) or 10 ng/mL TGFβ_1_ for 24 h followed by a further 24 h in the presence of 10 ng/mL IL-1β or vehicle (0.1 % BSA in PBS). Conditioned media was collected and the concentration of secreted proteins (sVCAM-1 (**a**), CX3CL1 (**b**), MCP-1 (**c**), IL-8 (**d**) and IL-6 (**e**)) was determined by a multiplex cytometric bead array. One representative experiment (*N* = 4 wells) from three independent experiments is shown. ***p < 0.001 (ANOVA)

As TGFβ_1_ appears to be a critical modifier of the pericyte immune phenotype, we sought to investigate its ability to attenuate or synergize with IL-1β, another pro-inflammatory cytokine. IL-1β alone significantly enhanced the basal secretion of sVCAM-1 (*p* < 0.001; Fig. 3a), CX3CL1 (*p* < 0.001; Fig. 3b), MCP-1 (*p* < 0.001; Fig. 3c), IL-8 (*p* < 0.001; Fig. 3d) and IL-6 (*p* < 0.001; Fig. 3e). TGFβ_1_ was found to significantly reduce IL-1β-induced sVCAM-1 (*p* < 0.001; Fig. 3a), CX3CL1 (*p* < 0.001; Fig. 3b) and MCP-1 (*p* < 0.001; Fig. 3c) secretions; however, it had no effect on IL-8 expression (*p* > 0.05, Fig. 3d). Furthermore, co-stimulation of pericytes with IL-1β and TGFβ_1_ showed a synergistic release of IL-6, well above that seen with IL-1β treatment alone (*p* < 0.001, Fig. 3e).

### TGFβ_1_ signals through SMAD2/3 transcription factors but does not affect NF-kB translocation

**Fig. 4:**
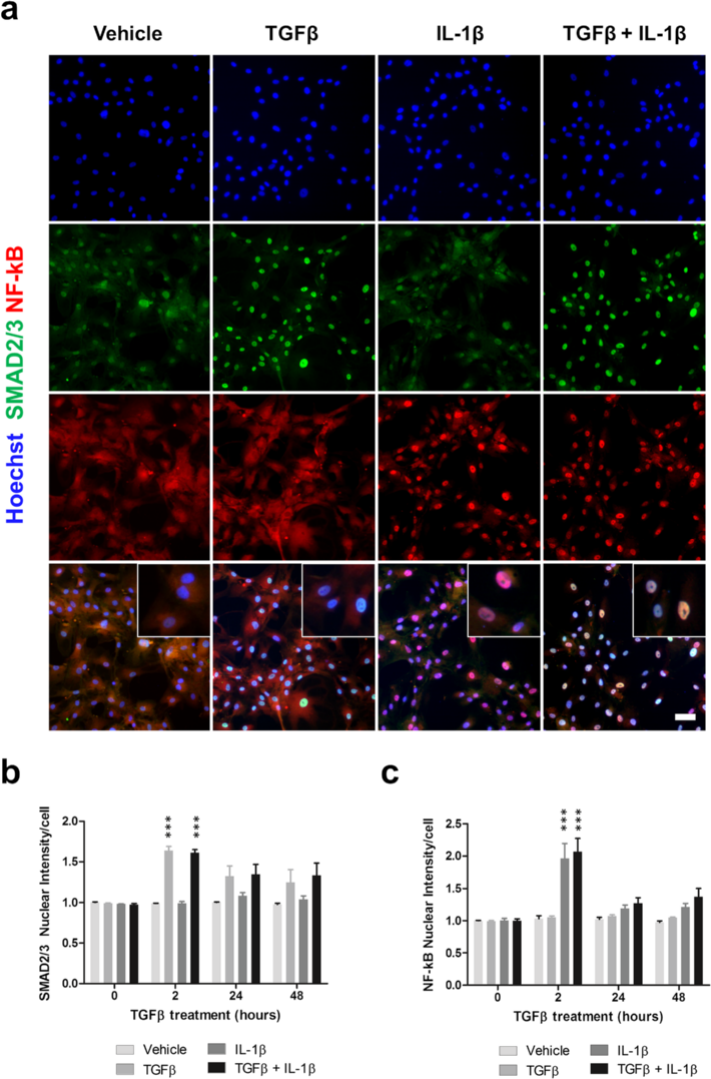
TGFβ_1_ signals through SMAD2/3 transcription factors but does not affect NF-kB translocation. Primary human brain pericytes were treated for 0–48 h with combinations of vehicle (1 mM citric acid, pH 3 with 0.1 % BSA), 10 ng/mL TGFβ_1_ and 10 ng/mL IL-1β. Cells were fixed and immunostained for NF-kB p65 and SMAD2/3. Representative images at 2 h are shown (**a**). The intensity of nuclear NF-kB (**b**) and nuclear SMAD2/3 (**c**) was determined by automated image analysis from four independent experiments. ***p < 0.001 versus vehicle control at each time point (two-way ANOVA). *Scale bar* = 50 μm

Inflammatory protein expression is largely controlled by transcription factor-mediated gene transcription. In order to further understand how TGFβ_1_ modifies the pericyte immune response, we investigated the ability of this cytokine to modify the localisation of two transcription factors, SMAD2/3 and nuclear factor kappa-light-chain-enhancer of activated B cells (NF-kB). Whilst SMAD2/3 showed a low level of basal nuclear localisation, TGFβ_1_ treatment significantly enhanced this at 2 h (*p* < 0.001; Fig. 4a, b), and remained slightly elevated (albeit not significantly) for up to 48 h (*p* > 0.05; Fig. 4a, b). IL-1β was found to have no effect on SMAD2/3 nuclear localisation at 2, 24 or 48 h (*p* > 0.05) alone and did not alter TGFβ_1_-induced nuclear localisation at any time point (*p* > 0.05; Fig. 4a, b). IL-1β significantly enhanced nuclear translocation of NF-kB at 2 h (*p* < 0.001) but not 24 h (*p* > 0.05) or 48 h (*p* > 0.05), whilst TGFβ_1_ has no effect at any time point (*p* > 0.05; Fig. 4a, c). Furthermore, TGFβ_1_ did not modify IL-1β-induced NF-kB translocation at 2 h (*p* > 0.05), 24 h (*p* > 0.05) or 48 h (*p* > 0.05; Fig. 4a, c).

### TGFβ_1_ attenuates phagocytosis by pericytes

**Fig. 5:**
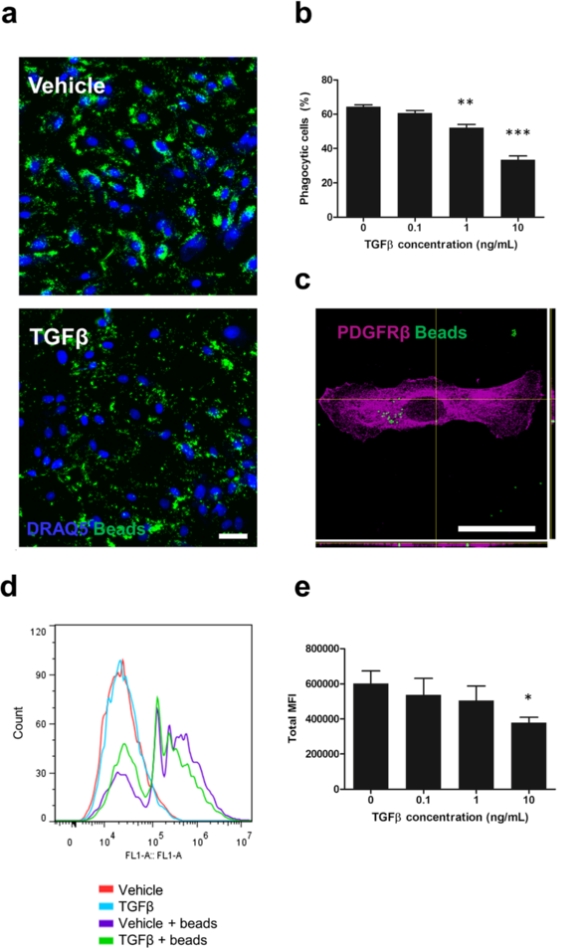
TGFβ_1_ attenuates phagocytosis by pericytes. Pericytes were treated for 24 h with 0–10 ng/mL TGFβ_1_ in 1 mM citric acid, pH 3 with 0.1 % BSA, followed by a further 24 h in the presence or absence of a 1:1000 dilution of fluorescent latex beads. Cells were fixed and nuclei counterstained with DRAQ5. Representative images for vehicle and 10 ng/mL TGFβ_1_ treatments are shown (**a**). The percentage of phagocytic pericytes was determined by automated image analysis from five independent experiments (**b**). Confocal microscopy of pericytes plated on glass coverslips, incubated for 24 h with a 1:10,000 dilution of fluorescent beads and immunostained with PDGFRβ confirmed internalisation of beads (**c**). Pericytes were treated for 24 h with 0–10 ng/mL TGFβ_1_ in 1 mM citric acid, pH 3 with 0.1 % BSA. For an additional two hours cells were incubated in the presence or absence of a 1:1000 dilution of fluorescent latex beads. Phagocytosis was determined by flow cytometry. One representative plot is shown (**d**) and the mean fluorescent intensity (MFI) of five independent experiments was determined (**e**). *p < 0.05, **p < 0.01, ***p < 0.001 versus vehicle control (ANOVA). *Scale bar* = 50 μm

Phagocytosis is a critical function of specific immune cells and is necessary to clear pathogenic substances from the brain [47]. Inflammatory cytokines, including TGFβ_1_, have previously been shown to modify the phagocytic ability of parenchymal brain cells, particularly microglia and astrocytes. Given the ability of TGFβ_1_ to modify pericyte inflammatory responses, we queried whether it could influence phagocytosis by these cells. TGFβ_1_ significantly attenuated pericyte phagocytosis of fluorescent latex beads at 1 ng/mL (*p* < 0.01) or 10 ng/mL (*p* < 0.001) as determined by an automated imaging-based assay (Fig. 5a, b). To confirm that beads were internalised and not simply bound to the cell membrane confocal microscopy was performed (Fig. 5c). A flow cytometry based assay was also employed to measure phagocytosis which again revealed a significant reduction in phagocytic ability by pericytes following a 10 ng/mL TGFβ_1_ treatment (*p* < 0.05, Fig. 5d, e) whilst lower concentrations trended towards a reduction.

### TGFβ_1_ reduces gene expression of scavenger receptors

**Fig. 6:**
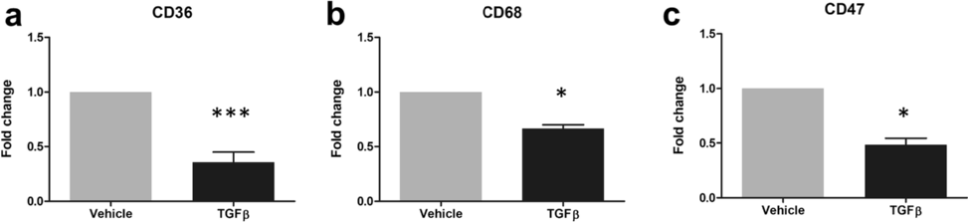
TGFβ_1_ reduces gene expression of scavenger receptors. Primary human brain pericytes were treated with vehicle (1 mM citric acid, pH 3 with 0.1 % BSA) or 10 ng/mL TGFβ_1_ for 24 h. RNA was extracted and expression of CD36 (**a**), CD68 (**b**) and CD47 (**c**) and determined by qRT-PCR from three independent experiments. *p < 0.05, ***p < 0.001 versus vehicle control (Student’s *t* test)

Scavenger receptors have a key role in recognising and coordinating the uptake of a wide range of macromolecules, including fluorescent beads and disease related proteins such as amyloid beta [48, 49]. As TGFβ_1_ was found to inhibit the phagocytic ability of pericytes, we investigated its ability to modify the expression of receptors involved in scavenging macromolecules. A 24-h treatment with TGFβ_1_ was found to significantly attenuate pericyte expression, as determined by qRT-PCR, of the receptors *CD36* (*p* < 0.001, Fig. 6a), *CD68* (*p* < 0.05, Fig. 6b) and *CD47* (*p* < 0.05, Fig. 6c), highlighting a possible mechanism behind TGFβ_1_-reduced phagocytic ability.

### TGFβ_1_ reduces pericyte proliferation but does not affect their viability

**Fig. 7:**
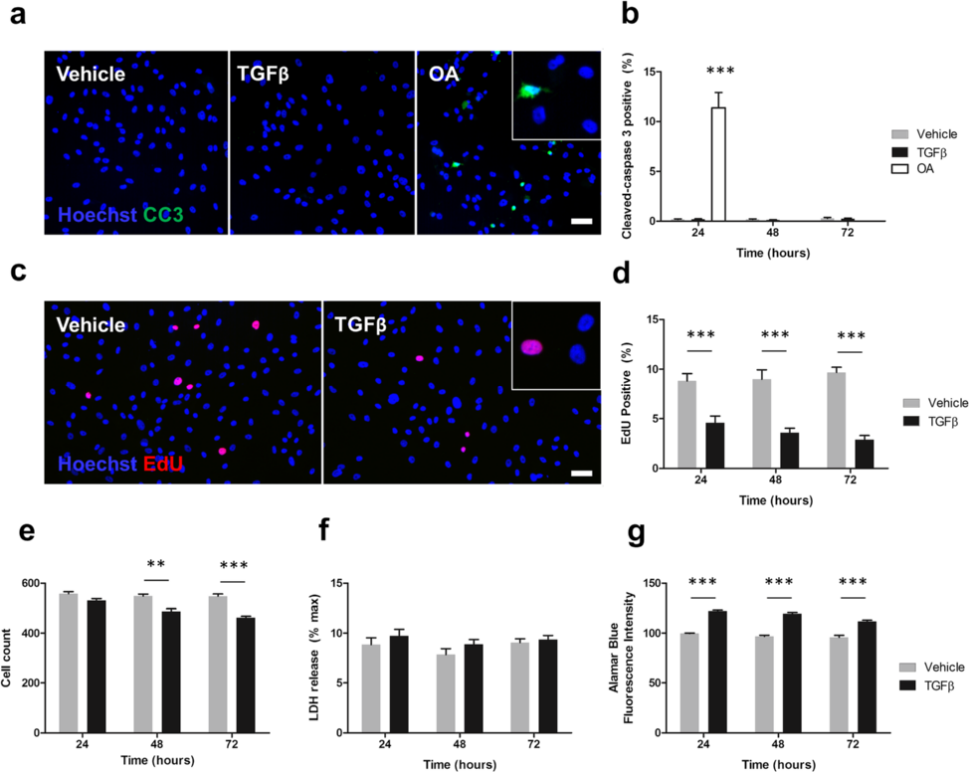
TGFβ_1_ decreases pericyte proliferation but does not affect their viability. Primary human brain pericytes were treated with vehicle (1 mM citric acid, pH 3 with 0.1 % BSA) or 10 ng/mL TGFβ_1_ for 0–72 h. Cells were fixed and immunostained for cleaved caspase 3 (CC3). Nuclei were counterstained with Hoechst. Cells treated for 24 h with 50 nM okadaic acid (OA) were used as a positive control. Representative images from the 24 h time point are shown (**a**). The percentage of CC3 positive cells was determined by automated image analysis from three independent experiments (**b**). Primary human brain pericytes were treated with vehicle (1 mM citric acid, pH 3 with 0.1 % BSA) or 10 ng/mL TGFβ_1_ for 0–72 h. For the final 24 h, cells were incubated with 10 μM EdU. Cells were fixed, EdU visualised and nuclei counterstained with Hoechst. Representative images from the 72-h time point are shown (**c**). The percentage of EdU positive cells (**d**), and the number of cells/field of view was determined by automated image analysis from three independent experiments (**e**). Primary human brain pericytes were treated with vehicle (1 mM citric acid, pH 3 with 0.1 % BSA) or 10 ng/mL TGFβ_1_ for 0–72 h. Cytotoxicity was determined from three independent experiments by an LDH release assay using cells lysed with DMEM/F12 containing 1 % Triton-X 100 as a positive control (**f**). Viability was determined from three independent experiments by a two hour treatment with Alamar Blue (**g**). *p < 0.05, **p < 0.01, ***p < 0.001 versus vehicle control for each time point (two-way ANOVA). *Scale bar* = 50 μm

Functional responses of cells, including phagocytosis, can be influenced by general changes in cell viability. In order to exclude the possibility that TGFβ_1_ modifies phagocytosis through compromising pericyte viability, we undertook a range of assays with respect to examining cell health. TGFβ_1_ was unable to stimulate pericyte apoptosis, as determined by antibody labelling and imaging of the early apoptotic marker cleaved caspase 3 (CC3) at any time point (*p* > 0.05; Fig. 7a, b). Due to the low level of basal pericyte apoptosis, validation of the CC3 antibody was determined by okadaic acid-induced apoptosis (Fig. 7a, b). TGFβ_1_ treatment did result in a significant reduction of EdU positive cells, indicative of reduced cell proliferation, at 24, 48 and 72 h (*p* < 0.001; Fig. 7c, d). However, these proliferative changes resulted in only small, albeit significant (48 h, *p* < 0.01; 72 h, *p* < 0.001), reductions in cell number (Fig. 7e). Cellular cytotoxicity, as determined by LDH release, revealed no change with TGFβ_1_ treatment at any time point (*p* > 0.05; Fig. 7f) whilst an Alamar Blue® viability assay showed an increase at 24, 48 and 72 h (*p* < 0.001; Fig. 7g).

## Discussion

TGFβ_1_ is a pleotropic cytokine that has been widely studied with respect to CNS inflammatory responses. Due to a ubiquitous anti-inflammatory microglial phenotype in response to TGFβ_1_ stimulation, several studies have suggested utilisation of TGFβ_1_ in limiting brain inflammation [10, 26–28]. However, microglia are not the only immunologically active parenchymal brain cell, and therefore, investigating the effect of TGFβ_1_ treatment on other cell types is warranted. Several groups, including our own, have shown that human brain pericytes can produce inflammatory responses in vitro and that these responses do not necessarily mimic microglial responses [27, 50].

To investigate transcriptome-wide changes in inflammatory mediators, microarray analysis of human brain pericytes treated with TGFβ_1_ was first performed. Whilst several inflammatory mediators displayed an attenuated expression as expected, a number of inflammatory genes were also found to be up-regulated following TGFβ_1_ stimulation. Eight genes were chosen for qRT-PCR validation consisting of four up-regulated genes (*IL6*, *MMP2*, *NOX4* and *COX2*) and four down-regulated genes (*VCAM1*, *IL8*, *MCP1* and *CX3CL1*). As observed previously, qRT-PCR data correlated strongly with microarray analysis, with all genes examined showing consistent trends [37]. In order to confirm mRNA changes at the protein level, a cytometric bead array was employed to measure soluble and secreted concentrations of the aforementioned cytokines, chemokines and adhesion molecules. With the exception of IL-8, TGFβ_1_ was found to modify inflammatory mediator secretions in a manner consistent with microarray and qRT-PCR data. Furthermore, when co-stimulated with TGFβ_1_ and the pro-inflammatory cytokine IL-1β, IL-6 expression was synergistically induced whilst MCP-1, CX3CL1, VCAM-1 and MCP-1 all demonstrated reduced secretions.

Like TGFβ_1_, the function of IL-6 in the brain has been widely studied but is not well defined. In certain instances, IL-6 appears to act as a pro-inflammatory cytokine further enhancing CNS immune responses [51, 52]. Paradoxically, IL-6 also appears to have anti-inflammatory functions and can aid neuronal survival in the presence of inflammation [51, 53]. Similarly, COX-2 induction has been widely debated with regards to its inflammatory phenotype. Whilst initially perceived to have a solely pro-inflammatory function, many of the COX-2-derived prostaglandins can have anti-inflammatory effects in a cell- and context-dependent manner [54–56].

MMP-2 is a matrix metalloproteinase involved in the breakdown of extracellular matrix (ECM) proteins, including type IV collagen, a major constituent of the basement membrane [57]. MMP-2 expression in the vasculature promotes BBB breakdown through reduced basement membrane expression and endothelial tight junction deregulation, subsequently enhancing leukocyte infiltration [58–60]. MMP-2 also facilitates cancer cell invasion in glioblastoma multiforme, a brain tumour in which TGFβ_1_ is highly expressed [61–63]. Furthermore, MMP-2 can cleave non ECM substrates, including latent TGFβ_1_ which can promote an increased release of its active form, potentially creating a positive feedback loop [64]. TGFβ_1_-enhanced MMP-2 expression has been recently observed in brain pericytes, corroborating our data [65]. NOX4 is a member of the NADPH oxidase family and contributes to cellular superoxide production. NOX family members are highly expressed in professional phagocytic cells and reactive oxidative species (ROS) are crucial in the killing of micro-organisms [66]. However, NOX-derived ROS have also been implicated in arteriosclerosis, disturbance of vascular tone, disruption of the BBB and endothelial cell apoptosis through oxidative stress [67–69]. NOX4 induction is a characteristic feature in the vasculature, showing expression in the endothelium [70, 71], vascular smooth muscle cells [72, 73] and more recently human brain pericytes [74]. TGFβ_1_-mediated MMP-2 and NOX4 induction may therefore have a significant impact in the disruption of CNS vasculature following injury.

Cytometric bead array was also performed to examine soluble VCAM-1, MCP-1, IL-8 and CX3CL1 secretions. Whilst VCAM-1, MCP-1, and CX3CL1 all showed attenuated secretions with TGFβ_1_, the level of IL-8 was not significantly altered. A similar response has been observed previously in our lab whereby changes in IL-8 mRNA did not correlate with IL-8 secretions [42]. The reasons for this discrepancy are currently unclear.

VCAM-1 is an adhesion molecule expressed by cells of the vasculature, including endothelial cells and pericytes [40, 75]. Unlike the related adhesion molecule intracellular adhesion molecule 1 (ICAM-1), VCAM-1 was found to be expressed under basal conditions by human brain pericytes [42]. VCAM-1 aids the adhesion and subsequent transmigration of leukocytes across brain vasculature [76]. This interaction occurs via binding of very late antigen-4 (VLA-4), an integrin which undergoes conformational changes to allow VCAM-1 mediated adhesion after chemotactic activation [75–78]. Whilst typically implicated in endothelial-immune cell interactions, pericytes can also control immune cell adhesion and migration across the BBB [40, 79]. MCP-1 is a monocyte chemokine that attracts peripheral immune cells through a concentration-dependent gradient. Elevated MCP-1 expression is observed in the brain following an insult and chemoattracts parenchymal microglia as well as peripheral circulating leukocytes to the injured site, thereby contributing to a pro-inflammatory CNS phenotype [80–82]. Attenuated VCAM-1 and MCP-1 expression could therefore represent an anti-inflammatory function of TGFβ_1_ through limiting peripheral immune cell infiltration.

Fractalkine (CX3CL1) is a unique protein which functions as an adhesion molecule when membrane-bound, or a leukocyte chemokine in its soluble form [83–86]. Like MCP-1 and VCAM-1, attenuated expression of this mediator could thereby limit peripheral immune cell CNS entry. However, the effects of CX3CL1 are exerted specifically through the fractalkine receptor (CX3CR1) and whilst circulating leukocytes express this receptor [83, 87], in the brain, CX3CR1 expression is limited predominantly to microglia [88]. CX3CL1-CX3CR1 interactions in the CNS promote a strong anti-inflammatory microglial phenotype, whilst CX3CR1 knockout mice showed microglial-mediated neurotoxicity [88–90]. Fractalkine signalling therefore appears to be vital in controlling microglial inflammatory responses. CX3CL1 is believed to be expressed predominantly by neurons in the CNS [90], and to our knowledge, this is the first evidence of fractalkine secretion by brain pericytes and this highlights a possible role for these cells in modulating microglial inflammation. TGFβ_1_-attenuated CX3CL1 expression in pericytes may therefore deregulate microglial-mediated immune responses.

Modifications of cellular inflammatory responses are largely controlled through transcription factor-mediated gene transcription. Consistent with previous literature, TGFβ_1_ produced a pronounced induction of nuclear SMAD2/3 [91, 92]. However, it failed to induce nuclear translocation of NF-kB alone or modify the IL-1β-induced localisation of NF-kB. Previous studies, albeit in different cell types, report both TGFβ_1_-mediated NF-kB translocation or lack thereof [93, 94]. This discrepancy highlights the cell-specific effects of TGFβ_1_. Indeed, cell type-dependent responses with TGFβ_1_ have been observed previously in our lab with respect to astrocyte and microglial inflammatory responses [27]. Surprisingly, although TGFβ_1_-induced SMAD2/3 nuclear localisation was largely absent after 24 h, the induction of several inflammatory markers approached their peak 72 h after TGFβ_1_ treatment. It is therefore unlikely that SMAD2/3-modified gene transcription was directly involved in stimulating certain TGFβ_1_-induced inflammatory changes. Instead, it is possible that the early induction of select markers, particularly NOX4, could influence the expression of other inflammatory proteins. Indeed, pericyte-derived NOX4 has been previously implicated in BBB breakdown through enhanced neuroinflammatory responses, including MMP-9 induction [95]. Whilst it is currently unclear precisely how TGFβ_1_ modifies pericyte inflammatory responses, it appears that it is not through the prototypical inflammatory transcription factor NF-kB. Further studies examining a role for SMAD transcription factors in pericyte immune responses are therefore required

Aside from their role in expressing inflammatory mediators, brain pericytes also possess phagocytic ability with respect to latex beads and other particulate matter [41, 96]. Using confocal microscopy, we confirmed that our pericyte cultures were capable of phagocytosis in vitro and this function was able to be quantitatively determined by microscopy- and flow cytometry-based assays. TGFβ_1_ was found to significantly reduce the phagocytic uptake of latex beads in a concentration-dependent fashion. Interestingly, enhanced expression of TGFβ_1_ in the brain significantly elevates fibrillar amyloid beta deposition, exclusively in the vasculature [29, 97]. Our results suggest that TGFβ_1_-attenuated phagocytic function could contribute to the increased vasculature-associated amyloid. Due to their phagocytic ability and anatomical location, it is possible that pericytes have a key role in clearing pathogenic substances from the brain parenchyma. Indeed, pericytes have recently been shown to internalise amyloid beta, whilst their loss accelerates cerebral amyloid angiopathy [98]. Despite showing phagocytic capability, it is important to note that their rate of uptake was notably lower than that of microglia, the brains resident macrophage [99].

Whilst it is currently unclear precisely how TGFβ_1_ reduced pericyte phagocytic function, it was found to decrease the expression of the scavenger receptors CD36, CD47 and CD68. These receptors recognise a range of macromolecules, including latex beads and amyloid beta, and stimulate their internalisation [100, 101]. Inhibition of CD36 and CD47 can prevent phagocytic function [48, 102, 103] whilst TGFβ_1_ has previously been shown to attenuate CD36 expression in macrophages [104, 105].

As well as specific receptor involvement, alterations in phagocytosis may be achieved simply by modifying cell viability. Due to the range of inflammatory mediators induced by TGFβ_1_, this was certainly a possibility. Indeed, TGFβ_1_-induced NOX4 has been shown to precipitate endothelial cell apoptosis through oxidative stress [106]. In order to exclude a generalised loss of pericyte viability in this phagocytic response, we examined the effect of TGFβ_1_ on pericyte health. Whilst a decrease in cell number was observed with TGFβ_1_ treatment, this was explained by a reduction in pericyte proliferation and not apoptotic or necrotic death or alterations in viability.

Aside from pericytes, several other cells of the neurovasculature unit, including microglia [25], astrocytes [11] and endothelial cells [106], contain TGFβR’s and may therefore contribute to TGFβ_1_-induced immune responses. Whilst TGFβ_1_ has been previously studied with respect to in vivo neurovasculature function, the ability of numerous cell types to respond to this growth factor compromises the ability to study cell type-specific functions, particularly secreted cytokines, chemokines and ROS production. Indeed in differing in vivo models, TGFβ_1_ has been shown to exacerbate BBB permeability through MMP-9 induction [107] as well as preventing BBB breakdown through MMP9 suppression [108]. Whilst understanding the role of TGFβ_1_ in each cell type is vital in understanding whole-organism changes, it should be emphasised that isolated pericyte cultures lack the paracrine signalling with other parenchymal brain cells present in vivo, and the functional outcomes of TGFβ_1_ expression in the neurovasculature could differ as a result.

## Conclusions

TGFβ_1_ attenuated the expression of key chemokines and adhesion molecules involved in CNS leukocyte trafficking and control of microglial function, as well as reducing their phagocytic ability. However, it also enhanced the expression of classical pro-inflammatory cytokines and enzymes which can disrupt BBB function. Together, these data suggest that TGFβ_1_ induction following brain injury stimulates a unique phenotype in brain pericytes which is neither specifically pro- nor anti-inflammatory. The functional in vivo outcome of TGFβ_1_-stimulation on brain pericytes is therefore difficult to predict. However, the reduction in pericyte proliferation, combined with elevated IL-6, MMP-2 and NOX4 expression, as well as attenuated phagocytic functioning, suggests a detrimental action of TGFβ_1_ on the neurovasculature.

## Additional files

Additional file 1: Table S1. List of antibodies used for immunocytochemistry. List of antibodies, suppliers and dilutions used for immunocytochemistry studies. (16.9 kb) Additional file 2: Table S2. List of primers used for qRT-PCR. List of primer sequences and amplicon sizes for qRT-PCR studies. (15.7 kb)
