# Supplementary material for: TGF-beta1 regulates human brain pericyte inflammatory processes involved in neurovasculature function
Source: J Neuroinflammation. 2016 Feb 11;13:37. doi: 10.1186/s12974-016-0503-0 (PMC4751726; doi:10.1186/s12974-016-0503-0)
Supplement: Additional file 1: Table S1. — List of antibodies used for immunocytochemistry. List of antibodies, suppliers and dilutions used for immunocytochemistry studies. (16.9 kb) [file 12974_2016_503_MOESM1_ESM.docx]

Additional file 1: Table S1. List of antibodies used for immunocytochemistry

| Antibody | Company | Catalogue # | Dilution |
| --- | --- | --- | --- |
| Mouse anti-SMAD2/3 | Santa Cruz | SC-133098 | 1:500 |
| Rabbit anti-NF-kB p65 | Santa Cruz | SC-372 | 1:500 |
| Rabbit anti-PDGFRβ | Cell Signaling | mAb3169 | 1:500 |
| Rabbit anti-cleaved caspase 3 | Cell Signaling | 9661L | 1:500 |
| Goat anti-mouse Alexa Fluor® 488 | Invitrogen | A11001 | 1:500 |
| Goat anti-rabbit Alexa Fluor® 594 | Invitrogen | A11012 | 1:500 |
| Donkey anti-rabbit Alexa Fluor® 647 | Abcam | ab150075 | 1:500 |
