# Supplementary material for: TGF-beta1 regulates human brain pericyte inflammatory processes involved in neurovasculature function
Source: J Neuroinflammation. 2016 Feb 11;13:37. doi: 10.1186/s12974-016-0503-0 (PMC4751726; doi:10.1186/s12974-016-0503-0)
Supplement: Additional file 2: Table S2. — List of primers used for qRT-PCR. List of primer sequences and amplicon sizes for qRT-PCR studies. (15.7 kb) [file 12974_2016_503_MOESM2_ESM.docx]

Additional file 2: Table S2. List of primers used for qRT-PCR

| Gene |  | Sequence | Amplicon Length | |  |
| --- | --- | --- | --- | --- | --- |
| *GAPDH* (h) | Fw | CATGAGAAGTATGACAACAGCCT | | 113 bp |  |
|  | Rv | AGTCCTTCCACGATACCAAAGT | |  |  |
| *IL6* (h) | Fw | TTCGGTCCAGTTGCCTTCTC | | 77 bp |  |
|  | Rv | TCTTCTCCTGGGGGTACTGG | |  |  |
| *IL8* (h) | Fw | CAGAGACAGCAGAGCACACA | | 70 bp |  |
|  | Rv | GTGAGATGGTTCCTTCCGGT | |  |  |
| *COX2* (h) | Fw | AGGGTTGCTGGTGGTAGGAA | | 76 bp |  |
|  | Rv | TCTGCCTGCTCTGGTCAATG | |  |  |
| *MCP1* (h) | Fw | CAGCCAGATGCAATCAATGCC | | 190 bp |  |
|  | Rv | TGGAATCCTGAACCCACTTCT | |  |  |
| *NOX4* (h) | Fw | GTTGGGGCTAGGATTGTGTCT | | 85 bp |  |
|  | Rv | TCGGCACATGGGTAAAAGGA | |  |  |
| *CX3CL1* (h) | Fw | ATTCTTTCCTGAGGCTGGGC | | 74 bp |  |
|  | Rv | GGTCTTGGAGGGCAGAGAAC | |  |  |
| *VCAM1* (h) | Fw | TTGACTTGCAGCACCACAGG | | 85 bp |  |
|  | Rv | TCGTCACCTTCCCATTCAGTG | |  |  |
| *MMP2* (h) | Fw | GCAGTGGGGGCTTAAGAAGA | | 77 bp |  |
|  | Rv | AGCTGGTTGGTTCATGCACT | |  |  |
| *CD36* (h) | Fw | AAAATGGGCTGTGACCGGAA | | 72 bp |  |
|  | Rv | TCCAAACACAGCCAGGACAG | |  |  |
| *CD47* (h) | Fw | TTGGGGTTCGCTCTTGGATC | | 90 bp |  |
|  | Rv | GTGGCATTGTCTAGGGCAGT | |  |  |
| *CD68* (h) | Fw | CACAGTCCTGCCACCACTAG | | 92 bp |  |
|  | Rv | TGGGCAGAACTGGTGAATCC | |  |  |
